# The Effect of Ageing on Phase Transformations and Mechanical Behaviour in Ni-Rich NiTi Alloys

**DOI:** 10.3390/ma17102420

**Published:** 2024-05-17

**Authors:** Jerzy Ratajski, Błażej Bałasz, Katarzyna Mydłowska, Mieczysław Pancielejko, Łukasz Szparaga

**Affiliations:** 1 Department of Biomedical Engineering, Faculty of Mechanical and Power Engineering, Koszalin University of Technology (KUT), ul. Śniadeckich 2, 75-453 Koszalin, Poland; lukasz.szparaga@tu.koszalin.pl; 2 Rapid Prototyping Center, Faculty of Mechanical and Power Engineering, Koszalin University of Technology (KUT), ul. Śniadeckich 2, 75-453 Koszalin, Poland; blazej.balasz@tu.koszalin.pl; 3 Department of Technical Physics and Nanotechnology, Faculty of Mechanical and Power Engineering, Koszalin University of Technology (KUT), ul. Śniadeckich 2, 75-453 Koszalin, Poland; mieczyslaw.pancielejko@tu.koszalin.pl

**Keywords:** NiTi, phase transformation, additive manufacturing, SLM, solution annealing, ageing

## Abstract

In this article, the results of research on a NiTi alloy with a high nickel content (51.7 at.%), produced using the additive technology SLM method and subjected to isothermal ageing after solution annealing, are presented. The study involved the determination of the sequence of phase transformations occurring using differential scanning calorimetry (DSC) and the determination of the temperature range of these transformations. In parallel, the phase composition was determined using the XRD method; the hardness and the Young’s modulus were also determined. The analysis of the DSC results obtained indicates the following characteristic features of the NiTi alloy, which change with ageing time: (1) During cooling (from +150 °C to −50 °C), the type of transformation changes from a one-step transformation after solution annealing to a two-step transformation after the ageing process over 1, 20, and 100 h at 500 °C; (2) during heating (from −50 °C to +150 °C) for all the samples, regardless of the ageing time, only a one-step transformation from martensite M(B19′) to austenite A(B2) is observed; (3) the temperature at which the transformation starts increases with the ageing time; (4) the width of the total temperature range of the transformation M(B19′) → A(B2) during heating changes from large (ΔT = 49.7 °C), after solution annealing, to narrow (ΔT = 19.3 °C and ΔT = 17.9 °C after 20 h and 100 h of ageing); and, most importantly, (5) a comparison with the literature data shows that, irrespective of the composition of the NiTi alloy and the manufacturing technology of the alloy samples (regardless of whether this was traditional or additive technology), a sufficiently long ageing process period leads to the occurrence of the martensite → austenite transformation in the same temperature range.

## 1. Introduction

Additive technologies are increasingly used in the manufacture of a wide variety of parts, from aerospace applications to biomedical engineering. They are used to produce complex, non-standard elements that would be labour-intensive to produce using traditional methods. In the area of biomedical engineering, it is anticipated that additive manufacturing (AM) will have a revolutionary impact on the manufacture of implants and tissue-engineering structures, resulting in a personalised approach to regenerative medicine that is tailored to the individual patient [1,2,3,4,5,6,7]. In the aeronautical and aerospace sectors, they are used to manufacture lightweight and high-strength components that meet stringent safety standards [8,9]. Additive technologies are evolving, and they create opportunities for applications in new areas. Their flexibility, fast operation, and ability to create non-standard components make them highly effective technologies. One example is the increasing use of additive technologies to produce components in NiTi alloy (nickel–titanium alloys—Nitinol) characterised by unique thermo-mechanical properties. Three-dimensional printing of NiTi alloy components opens the way to more advanced applications in various industries and biomedical engineering [10,11,12,13,14]. However, there are also technical challenges associated with 3D printing of NiTi alloys, such as control of the microstructure and mechanical properties, that require further research and technological development.

One of the phenomena that occur during the additive manufacturing of Nitinol products using the SLM method is the loss of nickel in the matrix [15,16,17,18,19,20] due to evaporation. The loss is dependent on the process parameters and results in a temperature shift of the martensite → austenite transformation towards higher temperatures, compared to transformations in the output powder. Hence, it is of key importance to establish the relationship between the SLM process parameters and the amount of evaporated nickel. Independently, the loss of nickel can be controlled in the matrix, and thus influence the temperature changes of phase transformations, by applying an appropriate post-process heat treatment. The treatment results in a separation of nickel-enriched secondary phases, such as Ni_4_Ti_3_, resulting in a reduction in the nickel content of the matrix [20,21,22,23,24,25,26,27]. Even a small fluctuation of 0.1 at.% in the composition of the alloy can significantly change the onset and end temperatures of phase transformations, which in turn determines the potential for the use of Nitinol in various smart structures in a wide range of industries and in biomedicine [28,29]. In other words, by controlling the composition of the alloy and its microstructure, it is possible to influence its functional temperatures, which determine the shape memory effect and the superelasticity of the alloy.

Nevertheless, the relationship between the nickel and titanium content in the output powder, the 3D printing process parameters and post-process heat treatment parameters, and the temperature at which phase transformations occur, as well as their sequence of occurrence, combined with the achievement of optimal mechanical properties of these alloys for a given application, still require intensive research. This paper presents studies that contribute to this important research area, carried out on NiTi alloy samples that are rich in Ni (51.7 at.% Ni), produced by means of additive technology using the SLM method. In particular, the relationship between the post-process heat treatment time and the martensite–austenite phase transformation temperature was investigated. The accompanying changes in hardness and the Young’s modulus were also investigated.

## 2. Materials and Methods

### 2.1. Material

Ni-Ti powder with the following composition: Ni (51.7 at.%) and Ti (48.3 at.%) was supplied by BIMO TECH Sp. z o.o. (Wrocław, Poland). The purpose of selecting the chemical composition of the powder, characterised by an excess of nickel, was to study the effect of the separation of secondary phases as a result of heat treatment after the SLM process on the change in phase transformation temperatures and on the change in hardness and the Young’s modulus.

Independently, the elemental composition of the alloy was determined using the EDX method, i.e., energy-dispersive X-ray spectrometry (EDX), carried out using a JEOL SEM LV 5500 scanning electron microscope (Jeol Ltd., Tokyo, Japan) and, in parallel, through a phase composition analysis using the XRD method. In addition, the temperature ranges of the phase transformations were determined by differential scanning calorimetry (DSC), covering the onset and end temperatures of the austenitic transformation [A_s_, A_f_]; the onset and end temperatures of the intermediate transformation, i.e., the R phase [R_s_, R_f_]; and the onset and end temperatures of the martensitic transformation [M_s_, M_f_].

### 2.2. SLM Process

The samples were produced using an ORLAS CREATOR^®^ machine (O. R. Laser technologie GmbH, Dieburg, Germany) for selective laser melting (SLM). In the SLM method, the coating arm spreads a layer of powder on the working platform. The laser beam scans the surface of the layer according to a computer model. An appropriate selection of the process parameters allows a specific volume of the powder to be completely melted. The cycle is repeated until a complete component is obtained. In the SLM process, the printing environment is an important factor. To prevent oxidation of the material being melted, the working chamber must be filled with a protective gas [30,31]. In this case, argon was used. Residual oxygen levels in the working chamber ranged from 0.05% to 0.1%. The post-production sample preparation included mechanical removal of supports and ultrasonic cleaning in distilled water.

### 2.3. Methods of Sample Characterisation

#### 2.3.1. Density

The density of the samples was measured using the Archimedes method, and the relative density was calculated from the formula ρ_r_ = ρ_e_/ρ_t_, where ρ_r_ is the relative density, ρ_e_ is the experimental density, and ρ_t_ is the theoretical density of 6.50 g/cm^3^.

#### 2.3.2. Differential Scanning Calorimetry, DSC

The DSC thermal method was used to determine phase transformation temperatures. The tests were carried out using a Netzsch DSC Polyma 214 device (NETZSCH-Gerätebau GmbH, Selb, Germany) in an argon atmosphere. The thermal cycling range tested was from −50 °C to +150 °C and from +150 °C to −50 °C, maintaining a heating–cooling rate of 10 °C/min.

#### 2.3.3. Phase Analysis

The phase composition of the NiTi samples was determined by X-ray diffraction (XRD) in the Bragg–Brentano geometry using an Empyrean diffractometer (Malvern Panalytical, Malvern, UK) with a Cu-Kα radiation source (λ = 1.5406 Å). HighScore Plus V. 4.0 software was used to analyse the diffractograms obtained, linked to the ICDD PDF 4+ 2023 diffraction image database.

#### 2.3.4. Sample Morphology

The morphology of the samples after the process was observed using a Thermo Fisher Dual Beam SCIOS II scanning electron microscope (Thermo Fisher Scientific Inc., Waltham, MA, USA) with an ETD secondary electron detector and a TEM to THEMIS microscope with a field emission gun (FEG) electron source. The observations were carried out in a scanning transmission mode (STEM) and TEM.

#### 2.3.5. Hardness and Young’s Modulus

The indentation measurements were carried out using a G200 nanoindenter from KLA-Tencor Corporation (Milpitas, CA, USA) fitted with a Berkovich diamond indenter. Load and indentation depth curves were obtained in a linear load growth mode. The loading and unloading times of the indenter during the measurement were 90 s. The tip radius of the Berkovich indenter was 0.2 µm. Calibration was carried out on fused silica. The hardness and Young’s modulus values were determined from indentation curves using the Oliver–Pharr model.

## 3. Results

### 3.1. Powder Characteristics

#### 3.1.1. Morphology

**Figure 1:**
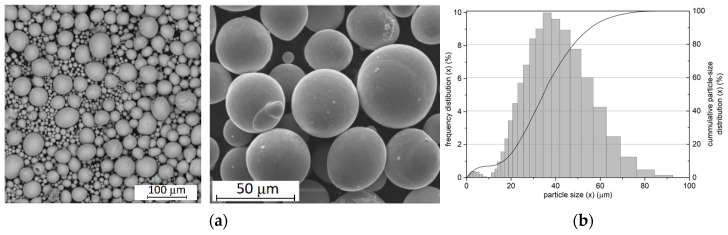
Powder morphologies and distribution: (**a**) powder morphology; (**b**) powder particle size distribution.

The surface morphology of the powders is shown in Figure 1. As can be seen, the powders are characterised by almost spherical shapes, with diameters in the range of 20–60 µm.

#### 3.1.2. Elemental Composition

**Figure 2:**
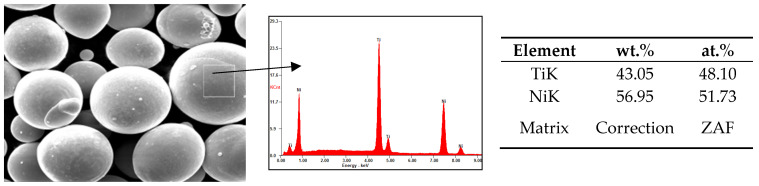
Elemental composition of powder determined using the EDX method.

The elemental composition determined using the EDX method (Figure 2) is consistent with the powder supplier’s certification.

**Figure 3:**
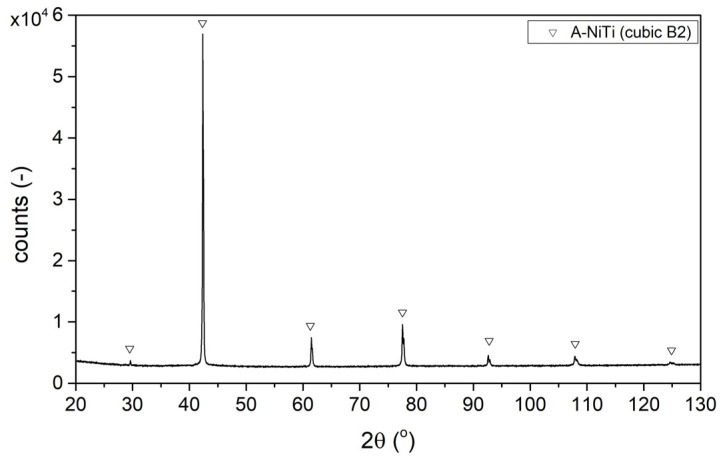
X-ray diffraction pattern observed in raw powder (austenite A).

**Figure 4:**
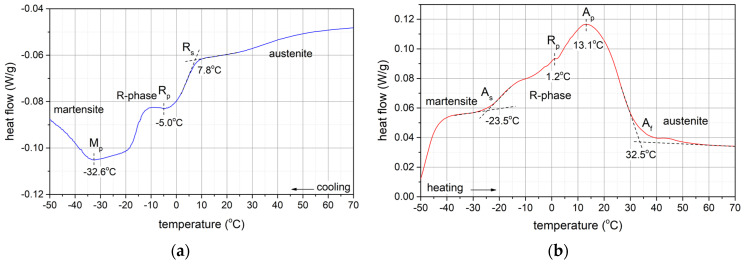
DSC (differential scanning calorimetry) curves of raw powder. (**a**) cooling of the sample, (**b**) heating of the sample.

In addition, the investigations of the phase composition using the XRD method (Section 3.1.3) and the phase transformation temperatures determined (Section 3.1.2) using differential scanning calorimetry qualitatively confirm the elemental composition of the powder. According to the literature [32], the powder with the elemental composition specified by the supplier, examined at room temperature using XRD, should possess an austenitic structure. Indeed, the analysis of the XRD diffractogram shown in Figure 3 confirms this phase structure of the powder. Also, the austenite–martensite and martensite–austenite phase transformations take place at temperatures below 0 °C (Figure 4), which is a characteristic property of the alloy with the elemental composition specified by the supplier.

#### 3.1.3. Phase Structure

The phase composition studies of the NiTi powder were carried out using the X-ray diffraction method in the Bragg–Brentano geometry using a Cu–Kα radiation source (λ = 1.5406 Å). A sample rotation with a period of 8 s was used to obtain a random grain distribution during the measurement. From the analysis of the diffractogram shown in Figure 3, it is evident that the powder has a crystalline structure and that all the diffraction peaks recorded are characteristic of the presence of a single phase, i.e., an intermetallic NiTi phase with a spatially centred cubic lattice (B2), i.e., austenite (γ-NiTi).

#### 3.1.4. Phase Transformations

The waveforms recorded (Figure 4) of heat flux changes using the differential scanning calorimetry (DSC) method are characterised by two exothermic peaks during the sample cooling from +150 °C as well as two endothermic peaks during the sample heating from −150 °C. During cooling, the first exothermic peak corresponds to the transformation of austenite A (B2) to the R phase, while the second exothermic peak observed at lower temperature is related to the transformation of the R phase to martensite M (B19′). Thus, the powder investigated shows a two-step transformation during cooling, according to the sequence A(B2) → R → M(B19′). During the heating process, a two-step transformation, M(B19′) → R → A(B2), was also observed, manifested by endothermic peaks. The crystal lattice of the R phase is a distortion of the austenite lattice, taking the form of a primitive hexagonal lattice (a rhombohedral structure). What acts as a catalyst for the B2 → R phase transformation is the presence of intermetallic secondary phases such as Ni_4_Ti_3_ [33,34,35,36,37,38,39,40].

**Figure 5:**
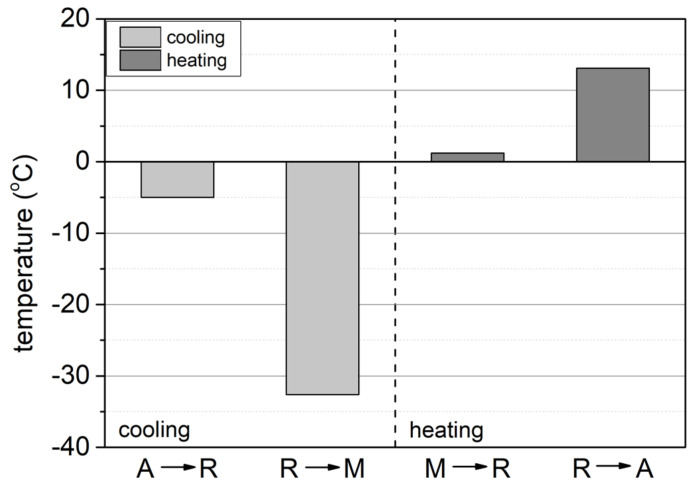
Characteristic temperatures at which the peaks representing a given transformation reach their maximum values.

In particular, the characteristic temperatures (Figure 5) at which the peaks representing a given transformation reach their maximum value are as follows:

- TR_max_ = −5 °C, A → R transformation during cooling;
- TM_max_ = −32.6 °C, R → M transformation during cooling;
- TR_max_ = 1.2 °C, M → R transformation during heating;
- TA_max_ = 13.10 °C, R → A transformation during heating.

### 3.2. Input–Output Correlation

#### 3.2.1. Input Parameters

The samples were fabricated by SLM using a process with the following parameters: laser power P = 186 W, scanning speed v = 1100 mm/s, line hatch spacing h = 0.08 mm, single layer thickness t = 0.03 mm. Based on the values of the input parameters used, the VED power density per unit volume of 70.45 J/mm^3^ was calculated.

$$VED=\frac{P}{v\cdot h\cdot t}$$

The VED parameter defines how much energy is released from a unit volume of the material during the SLM process. It can also be defined as a quantitative expression of thermal energy available for theof the material from the powder state to the dense state [26]. Samples of NiTi alloy after the process were subjected to solution annealing in a tubular furnace at a temperature of 1223 K for 0.5 h in an Ar atmosphere and then water-quenched.

#### 3.2.2. Sample Characteristics

The density of samples is one of the elementary parameters characterising samples after the additive manufacturing process, which is mainly determined by the presence of pores. The main cause of their formation in samples during the SLM process is gas entrapment. Density values can also be affected by cracks caused by high residual stresses. In the research presented in [41], it was established that a prerequisite for preventing pore formation is the use of relatively high laser power and appropriate values for the other SLM 3D printing parameters, ones that provide a relatively high VED to melt the powder in one layer and re-melt the previous layer to ensure epitaxial solidification.

In the study presented here, the density value obtained of the samples (ρ) determined according to the Archimedes law and the relative density value ρ_r_, i.e., related to the theoretical density value (ρ_t_ = 6.50 g/cm^3^), are ρ = 6.432 g/cm^3^ and ρ_r_ = 99.0%, respectively. This demonstrates that the selection of the process parameters, from the perspective of the density of the samples produced, is optimal.

**Figure 6:**
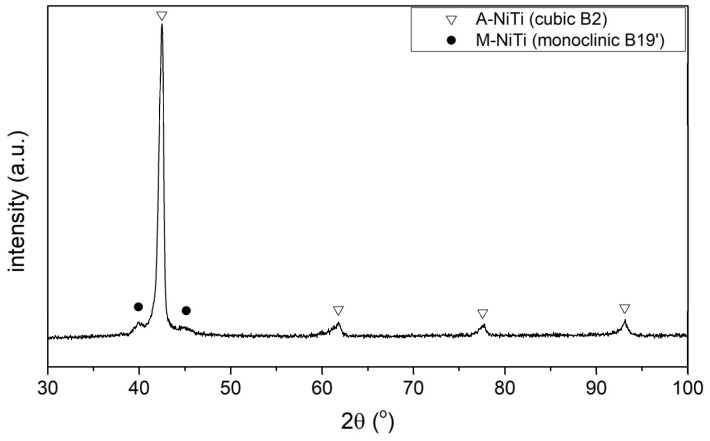
X-ray diffraction pattern of the sample.

Figure 6 shows an X-ray diffractogram of the sample after the SLM process and after solution annealing at 950 °C for 0.5 h. According to the diffractogram presented, the sample at room temperature is characterised by an austenitic–martensitic phase structure. The lines with the highest intensity come from the austenite NiTi phase, which proves its highest volume fraction. The appearance of low-intensity diffraction lines originating from the martensitic phase is evidence of a shift in the onset of the martensitic transformation towards higher temperatures in relation to the NiTi powder that the samples were made from, indicating a decrement in nickel during the incremental manufacturing process.

**Figure 7:**
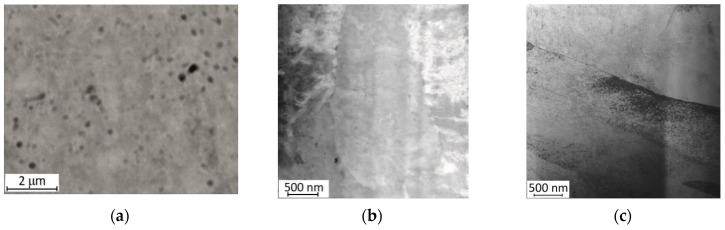
(**a**) SEM images of cross-sections of the sample in backscattered electron mode and (**b**,**c**) STEM images.

The microstructure of the samples was revealed by scanning electron microscopy in the backscattered electron mode and by transmission microscopy (Figure 7).

In the SEM image presented in Figure 7a, a relatively small number of pores can be observed, both spherical and irregularly shaped ones, whose dimensions do not exceed several dozen nanometres. These observations correspond with the relative density (ρ_r_ = 99%) determined, indicating that the pores occupy a small fraction of the sample’s volume. The STEM microphotographs seen in Figure 7b,c show grains with elongated shapes characteristic of the SLM method. This is a result of the crystallisation conditions and the cooling rate during the process.

The peaks recorded by differential scanning calorimetry, both exothermic ones during cooling and endothermic ones during heating, indicate a single-step phase transformation.

**Figure 8:**
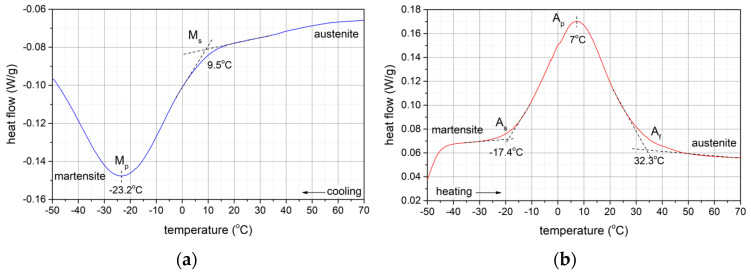
DSC (differential scanning calorimetry) curves of the sample. (**a**) cooling of the sample, (**b**) heating of the sample.

The temperatures (as depicted in Figure 8) at which the peaks corresponding to the maximum enthalpy change are observed signify the energy absorption during the endothermic transformation of martensite to austenite (A_p_) upon heating from a negative temperature and the energy release during the exothermic transformation of austenite to martensite (M_p_) upon cooling. These temperatures are as follows:

- TM_max_ = −23.2 °C: A → M transformation during cooling;
- TA_max_= 7 °C: M → A transformation during heating.

Compared to the phase transformations identified by DSC in the powder, the A(B2) → R and M(B19′) → R transformations disappear in the samples after the SLM process and dissolution annealing. This is due to the homogenisation of the phase composition of the samples, i.e., the dissolution of the secondary phases present in the powder, which is conducive to the formation of the R phase.

### 3.3. Isothermal Ageing

#### 3.3.1. Phase Composition Analysis

After the SLM process and dissolution annealing at 950 °C for t = 0.5 h, the samples were subjected to isothermal annealing (ageing) at 500 °C for 1, 20, and 100 h in an Ar atmosphere. The aim of these heat treatments was to produce Ni_4_Ti_3_ precipitates in the NiTi alloy with increased nickel content (51.7 at.%) and to study their effects on the sequence of phase transformations, on the characteristic transformation temperatures, and on the change in hardness and Young’s modulus. An interpretation of the phase and microstructural changes in the samples after a specific ageing time was carried out using complementary methods, i.e., differential scanning calorimetry (DSC) and X-ray diffraction (XRD).

In the additive manufacturing of NiTi alloy samples, differential scanning calorimetry (DSC) is the main method for monitoring phase transformation temperatures. First and foremost, it enables the determination of the temperature at which the austenitic (B2) phase transforms into the martensitic (B19′) phase and vice versa, which allows for the determination of the temperature range at which the shape memory effect is activated. This makes it possible to identify the area of potential applications of the unique functional characteristics of the NiTi alloy.

X-ray diffraction provides complementary information about the phase composition of samples. Among other things, this method makes it possible to identify secondary phases separated as a result of ageing at elevated temperatures.

**Figure 9:**
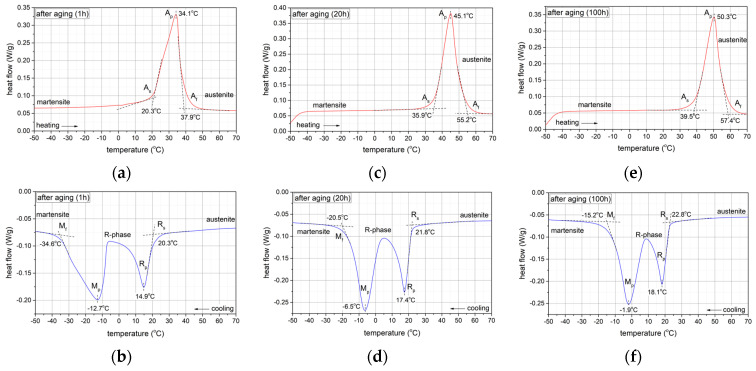
DSC (differential scanning calorimetry) curves of samples after (**a**,**b**) 1 h, (**c**,**d**) 20 h, and (**e**,**f**) 100 h ageing.

Figure 9 shows thermograms of the samples after specific ageing times, recorded using differential scanning calorimetry (DSC).

Figure 9 presents the thermograms of the samples obtained at specific ageing intervals utilizing differential scanning calorimetry (DSC). Following ageing periods of 1, 20, and 100 h, the phase transformations during cooling exhibit a consistent sequence: A(B2) → R → M(B19′), delineating a two-step transformation process. However, during the heating cycle, the formation of the R-phase is conspicuously absent in the thermograms, indicating a direct transformation from the martensitic phase to the austenitic phase: M(B19′) → A(B2). Additionally, it is noteworthy that all recorded phase transformations manifest a shift towards higher temperatures over the ageing intervals.

These findings suggest a unique transformation pathway in the studied material, characterized by a two-step transformation during cooling and a direct transformation from martensitic to austenitic phase during heating. Such behaviour could have significant implications for the material’s properties and its applications, particularly in fields where precise control over phase transformations is crucial, such as, for example, an implant or actuators.

**Figure 10:**
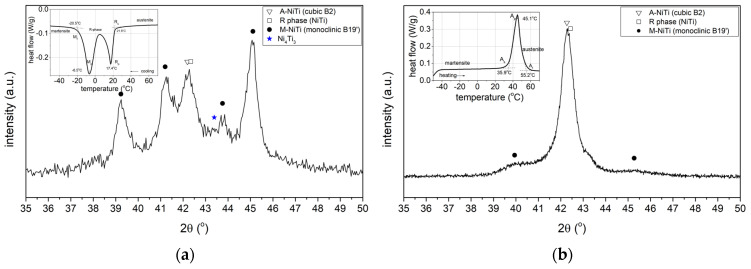
X-ray diffraction pattern of the sample (**a**) directly after 20 h ageing and (**b**) after ageing for 20 h and then cooled to −20 °C, and in the next step, heated to room temperature.

In order to gain additional knowledge of the phase transformation sequence, the samples were tested at room temperature by XRD after the ageing process (20 h, 500 °C) (Figure 10). One sample was tested immediately after the ageing process (Figure 10a), while the other sample was cooled to −20 °C after ageing and then heated to room temperature (Figure 10b).

**Figure 11:**
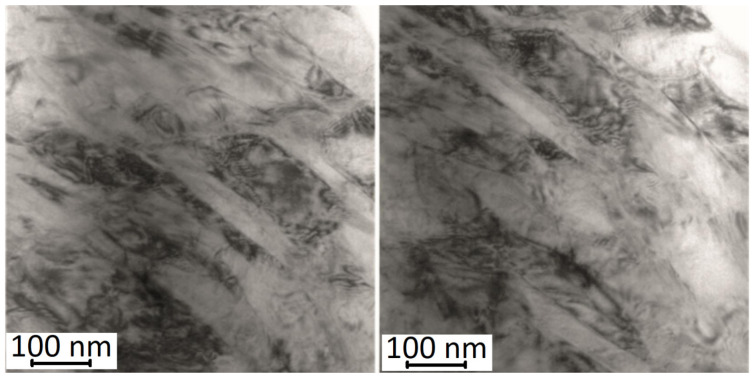
Martensite plates in NiTi alloy samples after ageing for 20 h at 500 °C.

In the case of a sample being immediately after ageing, the X-ray diffraction pattern (Figure 10a) shows diffraction peaks from the martensitic, austenitic, and rhombohedral phases R. Typical martensite plates for the alloy under study (Figure 11) were observed by transmission electron microscopy (TEM). These results confirm the analysis carried out with DSC demonstrating that a two-stage phase transformation occurs during cooling (Figure 9d).

In contrast, lines from the austenitic phase with high intensity and much smaller lines from the martensitic phase were recorded in the sample after ageing and cooling to a negative temperature (−20 °C) and then heating to room temperature (Figure 10b). These results also correspond with the DSC thermogram (Figure 9c) and indicate that during heating from a negative temperature, a one-step transformation from martensite to austenite occurs in the sample.

**Figure 12:**
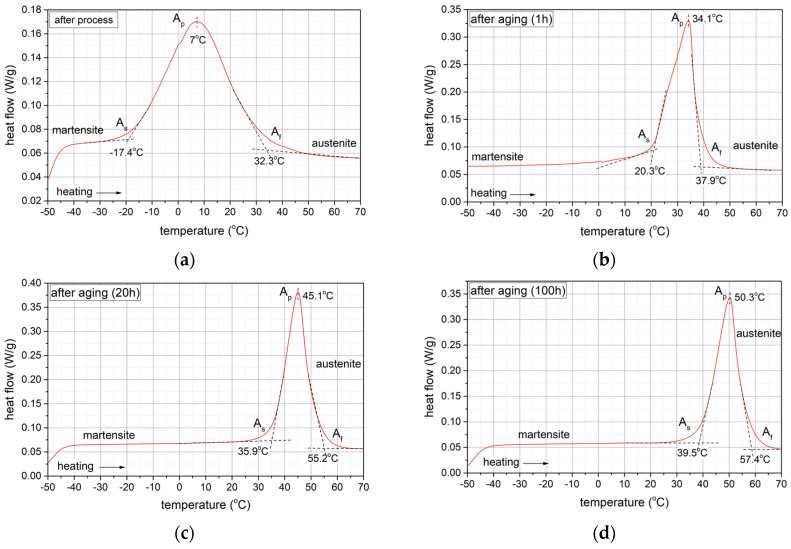
DSC thermograms during heating of NiTi samples (**a**) shortly after SLM process and after ageing at 500 °C (**b**) for 1 h, (**c**) for 20 h and (**d**) for 100 h.

**Figure 13:**
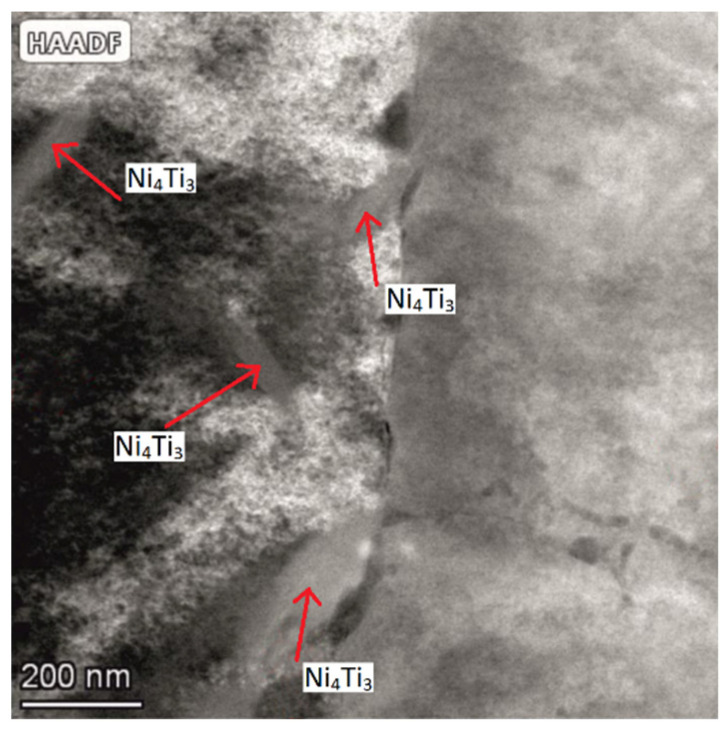
TEM micrographs of microstructure after the ageing process at T = 500 °C, t = 100 h.

Comparing the thermograms (DSC) shown in Figure 12, it can be seen that for the sample immediately after the SLM process, the characteristic peak associated with the formation of the austenitic phase in the sequence M(B19′) → A(B2) is much broader, i.e., the total temperature range of the M → A transformation is much greater, compared to aged samples. In addition, the onset (A_s_) and end (A_f_) temperatures of this transformation as well as the temperature at which the peak maximum (A_p_) occurs shift towards higher temperatures by several dozen degrees after ageing. This evolution of the phase transformation characteristics as a result of an increase in the ageing process time is related to changes occurring within the structure of the material. Above all, the Ni_4_Ti_3_, phases released during ageing, initially coherent with the matrix with increasing ageing time, become heterogeneous with the matrix [42]. Figure 13 shows a microscopic image (STEM) illustrating the presence of Ni_4_Ti_3_ phases after 100 h of ageing. The precipitations possess lenticular and disc-like shapes, and they reach dimensions of approximately 200 nm.

Secondary phases act as a catalyst for the formation of the pre-martensitic R-phase [33,34,35], while the shift in characteristic phase transformation temperatures towards higher temperatures is due to an increase in the number of secondary phases separated of the Ni_4_Ti_3_ type, binding more nickel than titanium and thus lowering the content of this element in the matrix [42,43].

In summary, the analysis of the DSC thermograms presented in the study, confirmed by XRD tests, indicates the following characteristic features of the Ni-rich NiTi alloy that change with the ageing time: (1) during cooling, the type of transformation changes from a one-step transformation after solution annealing to a two-step transformation after the ageing process over 1, 20, and 100 h; (2) when heating the samples from low temperatures for all of the ageing times, only a one-step transformation from M(B19′) to A(B2) is observed in the DSC thermograms; (3) the temperature at which thetransformation starts increases with the ageing time; and (4) the width of the total temperature range of the M → A transformation during heating changes from wide (ΔT = 49.7 °C, after solution annealing) to narrow (ΔT = 19.3 °C, after 20 h of ageing).

**Figure 14:**
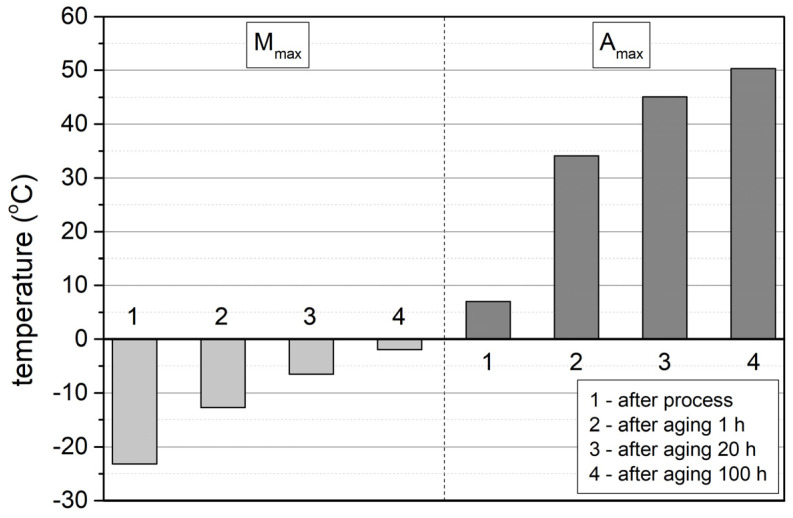
Temperatures at which peak maxima occur from B2 → B19′ (Mmax) and B19′ → B2 (Amax) transformations immediately following the SLM process and solution annealing (950 °C, t = 0.5 h) and after 1, 20, and 100 h of ageing, recorded in DSC thermograms.

The results presented for shorter ageing times differ from those presented in the key publication [42] on phase transformations in aged NiTi alloy samples. In the study involving samples made using the traditional method with a Ni content of 50.8 at.%, which had previously been aged for 1 and 10 h, only a sequence of two-step phase transformations, i.e., M(19′) → R → A(B2), was observed during heating using the DSC method. In contrast, a very good convergence of results was obtained after 100 h of ageing. In the compared studies, only a one-step transformation, M(B19′) → A(B2), was observed in the DSC thermograms during heating. Moreover, the peak maximum representing this transformation was practically recorded at the same temperature, i.e., TA_max_= 50 °C. It follows that, irrespective of the composition of the NiTi alloy and the manufacturing technology of this alloy sample, a sufficiently long ageing process time (in the cases analysed, at 500 °C for 100 h) leads to the occurrence of the martensite → austenite transformation in the same temperature range (Figure 14).

#### 3.3.2. Hardness and Young’s Modulus

**Figure 15:**
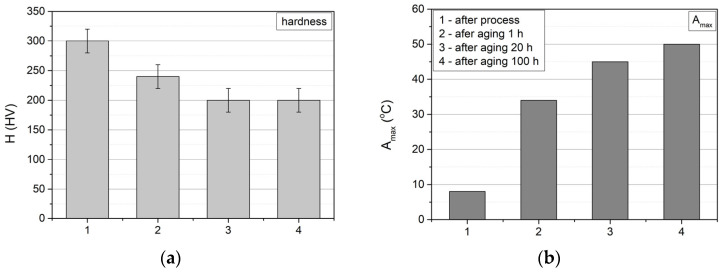
(**a**) Hardness of NiTi tubes and (**b**) the temperatures at which peak maximum (DSC) representing M(B19) → A(B2) transformation occurs.

The highest hardness value of the samples was measured after dissolution annealing, i.e., annealed at 950 °C for 0.5 h and cooled in water (Figure 15). Subsequently, it was observed that the hardness decreases during the ageing process. The greatest decrease occurs after one hour of ageing, from the value of 310 HV to 240 HV, and after 20 h, the hardness reaches the value of 200 HV, after which it does not change any further with increasing ageing time. The Young’s modulus remains practically unchanged during ageing, ranging from 52 to 54 MPa.

The results of the hardness changes correspond to the evolution of phase transformations in the alloys, as illustrated in Figure 15, where the hardness changes are compared with the temperature at which the maximum peaks of the M(B19′) → A(B2) transformation occur (TA_max_). As can be seen, the greatest changes both in hardness and in temperature occur after one hour of ageing. This is due to the fact that the NiTi alloy samples, after the dissolution annealing process, constitute a supersaturated Ni solution in the NiTi matrix, which has the highest hardness, and the ageing process generates the release of Ni_4_Ti_3_ secondary-phase particles, which are initially coherent with the matrix. With ageing, their dimensions increase and they become heterogeneous with the matrix. These processes cause a further decrease in the hardness and depletion of the matrix in Ni, resulting in a shift inthe M → A transformation towards higher temperatures.

## 4. Conclusions

Phase transformation temperatures, especially those at which A(B2) transforms into M(B19′) directly or via the R-phase during cooling and reversetransformation temperatures during heating when M(B19′) transforms into A(B2), are of crucial importance mainly from the technological perspective, as they determine at what temperature the shape memory effect is activated. To date, significant advances have been made in the knowledge of the properties of shape memory NiTi alloys, which enabled the development of diverse and interesting applications [44,45]. In the research on NiTi alloys, a lot of attention has been paid to multistage martensitic transformations. Among others, in the studies [17,18,42], it was pointed out that the microstructure of NiTi alloys and, in particular, dislocation substructures and Ni_4_Ti_3_ precipitation, are among the factors that influence the thermodynamic and kinetic conditions of martensitictransformation processes. The studies presented in [43,46] on martensitic transformations using molecular dynamics simulations (MDs) led to the proposal of a mechanism for these transformations, which relates to the presence of stresses around coherent Ni_4_Ti_3_ precipitation. Results from high-resolution TEM confirm that the presence of these stresses promotes the formation of martensite [47,48,49,50,51]. The study by Khalil-Allafi et al. [42] explained the formation of two-steptransformation sequences as a result of changes in Ni concentrations due to Ni_4_Ti_3_ phase precipitation and differences in nucleation barriers between the R and B19′ phases.

The research described in this paper also focused on the role of secondary-phase precipitation in NiTi alloys with high Ni content (Ni = 51.7 at.%) in phasetransformation temperatures and the change in hardness and in the Young’s modulus. Phasetransformation temperatures were determined by differential scanning calorimetry (DSC) and independently by XRD. The test samples were fabricated using additive technology and the SLM method. After the SLM process, the samples were dissolution annealed at 950 °C for 15 min and cooled in water. They were then subjected to annealing (ageing) at 500 °C for 1, 20, and 100 h. Simultaneously with the determination of the phase transformation temperatures, the hardness and the Young’s modulus were measured. Observations of the microstructures were also carried out using TEM. The following conclusions were drawn based on the results obtained:

- During cooling, the type of transformation changes from one-step after solution annealing to two-step after ageing for 1, 20, and 100 h.
- During heating of the samples from low temperatures, for all the ageing times, only a one-step transformation from M(B19′) to A(B2) is observed in the DSC thermograms.
- The transformation temperature M(B19′) → A(B2) increases with the ageing time.
- The width of the total transformation temperature range M(B19′) → A(B2) during heating varies from wide (ΔT = 49.70 °C) after solution annealing to narrow (ΔT = 19.30 °C) after 20 h of ageing.
- The change in the hardness values as a result of the ageing process corresponds to the change in temperature, at which point the maximum peak reflecting the M(B19′) → A(B2) transformation is observed, i.e., the changes in hardness minimise after 20 h of ageing.
- Comparison with the literature data proves that, irrespective of the NiTi alloy composition and sample manufacturing technology, a sufficiently long ageing process time (in the cases analysed, at 500 °C) leads to the occurrence of the martensite → austenite transformation in the same temperature range.

Further investigation is warranted to comprehensively analyse the phase transformations following ageing processes in Ni-rich NiTi alloys. This entails exploring alloys with varying Ni content and delving into the associated microstructural alterations alongside changes in strength and microbiological properties. Such research endeavours promise to deepen our understanding of the complex interplay between alloy composition, microstructure, and mechanical and biological performance.

## Data Availability

Data are contained within the article.

## References

[1] Aimar A., Palermo A., Innocenti B. (2019). The role of 3D printing in medical applications: A state of the art. J. Healthc. Eng..

[2] Velasco-Hogan A., Xu J., Meyers M.A. (2018). Additive manufacturing as a method to design and optimize bioinspired structures. Adv. Mater..

[3] Sabahi N., Chen W., Wang C.H., Kruzic J.J., Li X. (2020). A review on additive manufacturing of shape-memory materials for biomedical applications. JOM.

[4] Yan Q., Dong H., Su J., Han J., Song B., Wei Q., Shi Y. (2018). A review of 3D printing technology for medical applications. Engineering.

[5] Ye J., Wilson D.A., Tu Y., Peng F. (2020). 3D-Printed Micromotors for Biomedical Applications. Adv. Mater. Technol..

[6] Ahangar P., Cooke M.E., Weber M.H., Rosenzweig D.H. (2019). Current biomedical applications of 3D printing and additive manufacturing. Appl. Sci..

[7] Yuan L., Ding S., Wen C. (2018). Additive manufacturing technology for porous metal implant applications and triple minimal surface structures: A review. Bioact. Mater..

[8] Sacco E., Moon S.K. (2019). Additive manufacturing for space: Status and promises. Int. J. Adv. Manuf. Technol..

[9] Ishfaq K., Asad M., Mahmood M.A., Abdullah M., Pruncu C. (2022). Opportunities and challenges in additive manufacturing used in space sector: A comprehensive review. Rapid Prototyp. J..

[10] Lu H.Z., Ma H.W., Luo X., Wang Y., Wang J., Lupoi R., Yin S., Yang C. (2021). Microstructure, shape memory properties, and in vitro biocompatibility of porous NiTi scaffolds fabricated via selective laser melting. J. Mater. Res. Technol..

[11] Zhang Y., Attarilar S., Wang L., Lu W., Yang J., Fu Y. (2021). A review on design and mechanical properties of additively manufactured NiTi implants for orthopedic applications. Int. J. Bioprinting.

[12] Chmielewska A., Dobkowska A., Kijeńska-Gawrońska E., Jakubczak M., Krawczyńska A., Choińska E., Jastrzębska A., Dean D., Wysocki B., Święszkowski W. (2021). Biological and corrosion evaluation of in situ alloyed NiTi fabricated through laser powder bed fusion (LPBF). Int. J. Mol. Sci..

[13] Xu Z., Guo Y., Liu Y., Jia B., Sha P., Li L., Yu Z., Zhang Z., Ren L. (2023). An extremely efficiency method to achieve stable superhydrophobicity on the surface of additive manufactured NiTi Alloys: “UltrasonicFluorination”. Appl. Surf. Sci..

[14] Habijan T., Haberland C., Meier H., Frenzel J., Wittsiepe J., Wuwer C., Greulich C., Schildhauer T.A., Köller M. (2013). The biocompatibility of dense and porous nickel–titanium produced by selective laser melting. Mater. Sci. Eng. C.

[15] Chekotu J.C., Goodall R., Kinahan D., Brabazon D. (2022). Control of Ni-Ti phase structure, solid-state transition temperatures and enthalpies via control of L-PBF process parameters. Mater. Des..

[16] Ye D., Li S.F., Misra R.D.K., Zheng R., Yang Y.F. (2021). Ni-loss compensation and thermomechanical property recovery of 3D printed NiTi alloys by pre-coating Ni on NiTi powder. Addit. Manuf..

[17] Saedi S., Turabi A.S., Andani M.T., Haberland C., Elahinia M., Karaca H. (2016). Thermomechanical characterization of Ni-rich NiTi fabricated by selective laser melting. Smart Mater. Struct..

[18] Saedi S., Turabi A.S., Andani M.T., Haberland C., Karaca H., Elahinia M. (2016). The influence of heat treatment on the thermomechanical response of Ni-rich NiTi alloys manufactured by selective laser melting. J. Alloys Compd..

[19] Feng B., Wang C., Zhang Q., Ren Y., Cui L., Yang Q., Hao S. (2022). Effect of laser hatch spacing on the pored efects, phase transition and properties of selective laser melting fabricated NiTi shape memory alloys. Mater. Sci. Eng. A.

[20] Khoo Z.X., Liu Y., An J., Chua C.K., Shen Y.F., Kuo C.N. (2018). A review of selective laser melted NiTi shape memory alloy. Materials.

[21] Allafi J.K., Ren X., Eggeler G. (2002). The mechanism of multistage martensitic transitions in aged Ni-rich NiTi shape memory alloys. Acta Mater..

[22] Zhou N., Shen C., Wagner M.X., Eggeler G., Mills M.J., Wang Y. (2010). Effect of Ni4Ti3 precipitation on martensitic transition in Ti–Ni. Acta Mater..

[23] Yao X., Amin-Ahmadi B., Li Y., Cao S., Ma X., Zhang X.P., Schryvers D. (2016). Optimization of Automated Crystal Orientation Mapping in a TEM for Ni_4_Ti_3_ Precipitation in All-Round SMA. Shape Mem. Superelast..

[24] Ma C., Gu D., Dai D., Xia M., Chen H. (2018). Selective growth of Ni_4_Ti_3_ precipitate variants induced by complicated cyclic stress during laser additive manufacturing of NiTi-based composites. Mater. Charact..

[25] Novák P., Pokorný P., Vojtěch V., Knaislová A., Školáková A., Čapek J., Kopeček J. (2015). Formation of Ni–Ti intermetallics during reactive sintering at 500–650 °C. Mater. Chem. Phys..

[26] Ma J., Franco B., Tapia G., Karayagiz K., Johnson L., Liu J., Arroyave R., Karaman I., Elwany A. (2017). Spatial control of functional response in 4D-printed active metallic structures. Sci. Rep..

[27] Elahinia M., Moghaddam N.S., Amerinatanzi A., Saedi S., Toker G.P., Karaca H., Bigelow G.S., Benafan O. (2018). Additive manufacturing of NiTiHf high temperature shape memory alloy. Scr. Mater..

[28] Bormann T., de Wild M., Beckmann F., Müller B. (2013). Assessing the morphology of selective laser melted NiTi-scaffolds for a three-dimensional quantification of the one-way shape memory effect. Proc. SPIE.

[29] Bormann T., Schumacher R., Müller B., Mertmann M., DeWild M. (2012). Tailoring selective laser melting process parameters for NiTi implants. J. Mater. Eng. Perform..

[30] Bose S., Ke D., Sahasrabudhe H., Bandyopadhyay A. (2018). Additive manufacturing of biomaterials. Prog. Mater. Sci..

[31] DebRoy T., Wei H.L., Zuback J.S., Mukherjee T., Elmer J.W., Milewski J.O., Beese A.M., Wilson-Heid A., De A., Zhang W. (2018). Additive manufacturing of metallic components–process, structure and properties. Prog. Mater. Sci..

[32] Horvay K.M., Schade C.T. (2018). Development of nitinol alloys for additive manufacturing. Mater. Sci. Technol..

[33] Zhu J., Wu H.H., Wu Y., Wang H., Zhang T., Xiao H., Wang Y., Shi S.Q. (2021). Influence of Ni_4_Ti_3_ precipitation on martensitic transformations in NiTi shape memory alloy: R phase transformation. Acta Mater..

[34] Zhao Y., Yu Z., Ren X., Wang Q., Zhang B., Chen J., Xu W., Ren S., Qu X. (2023). Exceptional fatigue resistant NiTi wire mediated by R-phase. Int. J. Fatigue.

[35] Wang X., Kustov S., Verlinden B., Van Humbeeck J. (2015). Fundamental development on utilizing the R-phase transition in NiTi shape memory alloys. Shape Mem. Superelast..

[36] Honarvar M., Konh B., Podder T.K., Dicker A.P., Yu Y., Hutapea P. (2015). X-ray diffraction investigations of shape memory NiTi wire. J. Mater. Eng. Perform..

[37] Feng B., Kong X., Hao S., Liu Y., Yang Y., Yang H., Cui L. (2020). In-situ synchrotron high energy X-ray diffraction study of micro-mechanical behaviour of R phase reorientation in nanocrystalline NiTi alloy. Acta Mater..

[38] Duerig T.W., Bhattacharya K. (2015). The influence of the R-phase on the superelastic behavior of NiTi. Shape Mem. Superelast..

[39] Tao C., Zhou G., Huang H., Fu C., Zheng B., Zuo X., Chen L., Yuan X. (2024). Abnormal superelastic cyclic behavior and stress-induced martensitic transformation mechanism of NiTi alloy with R phase. J. Mater. Res. Technol..

[40] Yang J., Heogh W., Ju H., Kang S., Jang T.S., Jung H.D., Jahazi M., Han S.C., Park S.J., Kim H.S. (2024). Functionally graded structure of a nitride-strengthened Mg_2_Si-based hybrid composite. J. Magnes. Alloys.

[41] Chekotu J.C., Groarke R., O’Toole K., Brabazon D. (2019). Advances in selective laser melting of nitinol shape memory alloy part production. Materials.

[42] Khalil-Allafi J., Dlouhy A., Eggeler G. (2002). Ni_4_Ti_3_-precipitation during aging of NiTi shape memory alloys and its influence on martensitic phase transitions. Acta Mater..

[43] Pandolfi G.S., Martins S.C., Buono V.T., Santos L.A. (2020). Precipitation kinetics of Ti_3_Ni_4_ and multistage martensitic transformation in an aged Ni–rich Ni–Ti shape memory alloy. J. Mater. Res. Technol..

[44] Jiang S.Y., Zhang Y.Q., Zhao Y.N., Liu S.W., Li H.U., Zhao C.Z. (2015). Influence of Ni_4_Ti_3_ precipitates on phase transformation of NiTi shape memory alloy. Trans. Nonferrous Met. Soc. China.

[45] Guo W., Feng B., Yang Y., Ren Y., Liu Y., Yang H., Yang Q., Cui L., Tong X., Hao S. (2022). Effect of laser scanning speed on the microstructure, phase transformation and mechanical property of NiTi alloys fabricated by LPBF. Mater. Des..

[46] Chaudhari R., Vora J.J., Parikh D.M. (2020). A review on applications of nitinol shape memory alloy. Recent Adv. Mech. Infrastruct. Proc. ICRAM.

[47] Safranski D., Dupont K., Gall K. (2020). Pseudoelastic NiTiNOL in Orthopaedic Applications. Shap. Mem. Superelast..

[48] Ataollahi S., Mahtabi M.J. (2021). Effects of precipitate on the phase transformation of single-crystal NiTi alloy under thermal and mechanical loads: A molecular dynamics study. Mater. Today Commun..

[49] Li Z., Xiao F., Chen H., Hou R., Cai X., Jin X. (2021). Atomic scale modeling of the coherent strain field surrounding Ni4Ti3 precipitate and its effects on thermally-induced martensitic transformation in a NiTi alloy. Acta Mater..

[50] Xu K., Luo J., Li C., Shen Y., Li C., Ma X., Li M. (2022). Mechanisms of stress-induced martensitic transformation and transformation-induced plasticity in NiTi shape memory alloy related to superelastic stability. Scr. Mater..

[51] Yan B., Zhang Y., Jiang S., Yu J., Sun D., Tang M. (2021). Mechanical properties and fracture mechanisms of martensitic NiTi shape memory alloy based on various thermomechanical-processing microstructures. J. Alloys Compd..

